# Indoor Air Quality in Buildings: A Comprehensive Review on the Factors Influencing Air Pollution in Residential and Commercial Structure

**DOI:** 10.3390/ijerph18063276

**Published:** 2021-03-22

**Authors:** Mehzabeen Mannan, Sami G. Al-Ghamdi

**Affiliations:** Division of Sustainable Development, College of Science and Engineering, Hamad Bin Khalifa University, Qatar Foundation, Doha, Qatar; mmannan@hbku.edu.qa

**Keywords:** indoor air pollution, residential indoor pollutants, office indoor pollutants, school indoor pollutants, influencing factors indoor

## Abstract

Worldwide people tend to spend approximately 90% of their time in different indoor environments. Along with the penetration of outside air pollutants, contaminants are produced in indoor environments due to different activities such as heating, cooling, cooking, and emissions from building products and the materials used. As people spend most of their lives in indoor environments, this has a significant influence on human health and productivity. Despite the two decades of indoor air quality (IAQ) research from different perspectives, there is still a lack of comprehensive evaluation of peer-reviewed IAQ studies that specifically covers the relationship between the internal characteristics of different types of building environments with IAQ to help understand the progress and limitations of IAQ research worldwide. Therefore, this review of scientific studies presents a broad spectrum of pollutants identified in both residential and commercial indoor environments, highlighting the trends and gaps in IAQ research. Moreover, analysis of literature data enabled us to assess the different IAQs in buildings located in different countries/regions, thus reflecting the current global scientific understanding of IAQ. This review has the potential to benefit building professionals by establishing indoor air regulations that account for all indoor contaminant sources to create healthy and sustainable building environments.

## 1. Introduction

Research on the urban population has confirmed that people spend more than 90% of their daily lifespan in indoor environments. Apart from residential indoor environments, people spend a large proportion of their time in offices, educational institutes, and other different commercial and industrial buildings. Specific research in North America has shown that adults tend to spend 87% of their time in buildings, and the remainder of their time is spent in vehicles (6%) and outdoors (7%) [1]. As people spend a majority of their time in indoor environments, exposure to indoor air pollutants has a significant impact on both human health and effectiveness in the workplace. However, research on air quality has mostly focused on the outdoors, whereas indoor air quality (IAQ) and its impacts have received considerably less attention until the last decade [2]. Recently, both scientists and the public have focused on risks associated with IAQ because research has established that indoor air is more contaminated than outdoor air [3]. Due to continuous changes in living style and the materials used in indoor environments, there have been significant changes in terms of the nature and complex compositions of indoor air pollutants, which opens up avenues that need to be investigated in detail.

### 1.1. Patterns of Time Spent Indoors

**Figure 1:**
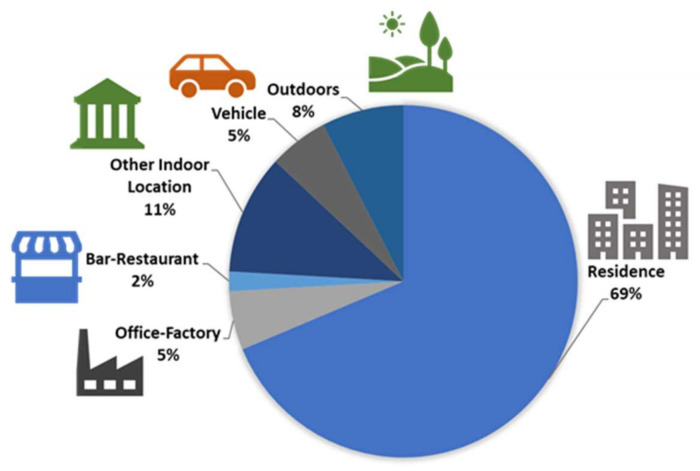
Pie chart of the percentage of time spent in indoor and outdoor environments. Data were collected from the United States Environmental Protection Agency (US EPA) sponsored National Human Activity Pattern Database (NHAPS). The total number of participants was 9196, and approximately 87% of the time spent in indoor environments was in residential buildings, office buildings, restaurants, and other indoor places, such as malls, stores, schools, churches, public building, salons, health clubs, parking garages, auto-repair shops, and laundromats [1].

Different life activities cause people to spend a majority of their time in different types of buildings, including residences, offices, schools, and restaurants (Figure 1). The air quality in indoor environments is a significant determinant of human health and wellbeing. Several studies have established links between positive human health impacts and improved indoor environments [4,5,6]. Low IAQ results in unwanted health conditions, including death in the worst-case scenarios. This highlights the importance of the IAQ of any indoor space where humans spend a majority of their time.

To better understand the factors affecting overall IAQ, the assessments of IAQ should be based on different types of buildings. Therefore, it is essential to understand the relation between IAQ and different residential and commercial buildings because building codes and regulations vary based on the type and end purpose of the building.

### 1.2. Indoor Pollution Sources and Health Impacts

The energy crisis of the 1970s introduced the importance of energy savings in buildings, which ultimately led to more airtight and insulated buildings worldwide [7]. For energy savings, reduced amounts of fresh air are circulated in air conditioning systems. Moreover, with the advent of improved living standards, more synthetic materials and chemicals are being used in buildings for indoor construction and decorating purposes. Additionally, pesticides, cleaning agents, air fresheners, and gases from cooking are other sources of indoor air pollution.

Because inadequate ventilation, lack of air conditioning systems, human activities, and numerous materials, chemicals, and gases mainly influence indoor pollution, different organizations, such as the United States Environmental Protection Agency (US EPA) and World Health Organization (WHO) have recognized IAQ as a multi-disciplinary phenomenon and classified pollutants into several categories. According to the WHO, in the year 2000, over 1.5 million deaths were caused by indoor air pollution [8]. Moreover, indoor air pollution has been recognized as the third main reason for disability-adjusted life years worldwide [9]. Table 1 provides some pollutants in indoor environments and their health impacts.

### 1.3. Purpose of Study

With the continuous growth of the population and economy, demand for high quality of life has introduced different new elements in indoor building environments. Additionally, different types of buildings are changing with time, which has an impact on IAQ and human health. Therefore, it is of utmost importance to analyze the IAQ in diverse end-use buildings to determine each possible indoor pollutant in specific kinds of buildings that are responsible for adverse health impacts. Despite the two decades of IAQ research from different perspectives, there is still a lack of organized evaluation of peer-reviewed IAQ studies that specifically cover both residential and commercial building environments. These would help to understand the factors influencing IAQ in different types of building environments along with the capacity to highlight the progress and limitations of IAQ research worldwide. A wider understanding of the relation between different building characteristics and air pollutant concentrations is required to enable possible sustainable solutions for better IAQ.

To address this gap, we reviewed scientific studies that focused on both residential and commercial building IAQ in different parts of the world. Therefore, the trends and gaps in scientific research for both the residential and commercial sectors that focus on quantitative changes in air parameters due to IAQ have been identified in this review. Moreover, we reviewed the internationally recognized IAQ standards and the sampling techniques applied in peer-reviewed studies. The breadth of this review was undertaken to support and accelerate future research on the design of optimal building environments to provide the best possible IAQ benefits for future healthy indoor spaces.

To achieve these objectives, this review includes scientific studies from different relevant scientific databases. The remainder of this review paper is structured under four major headings that discuss (1) international IAQ standards and assessment methods, (2) residential buildings and IAQ assessment, (3) commercial buildings and IAQ assessment, and (4) the conclusions and future scope.

## 2. Methodology

**Figure 2:**
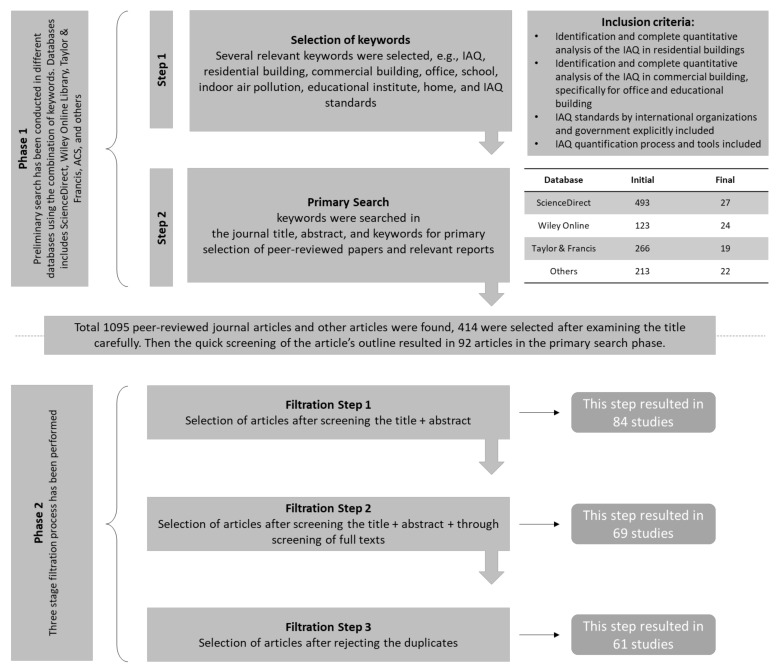
Literature search criteria and steps.

This review is formulated based on peer-reviewed journal articles from several renowned databases, such as ScienceDirect, Wiley Online Library, and Taylor & Francis. We mostly focus on papers published in the last twenty years to realize the periodical progress in scientific research. A few journal papers from other databases are also reviewed here (such as ACS), as mentioned in Figure 2. As this review considers the IAQ of both residential and commercial buildings, the home spaces where people spend majority of their time, as well as IAQ in two different commercial buildings (offices and educational institutes), have been reviewed. Other types of commercial buildings, such as hospitals, malls, and restaurants, are beyond the scope of this review because the percentage of time spent in these spaces is insignificant as compared to that spent in offices and educational institutes. In this review, we attempt to describe the progressive trend of IAQ research around the world; therefore, peer-reviewed journals across the world were considered. Along with the journal papers, a few conference papers and government reports were also analyzed to enhance the quality of the review.

A database search was completed using several combinations of keywords, e.g., IAQ, residential building, commercial building, office, school, indoor air pollution, educational institute, home, and IAQ standards. These keywords were searched in the journal title, abstract, and keywords for primary selection of peer-reviewed papers (phase 1 in Figure 2). A total of 1095 peer-reviewed journal articles and other papers, and reports were returned after the primary search from where 414 articles were selected after the careful screening of the article titles, keywords, and quick screening of the article’s outline. Screening in phase 1 has been done to eliminate those articles that are qualitative in nature and focus on IAQ for other types of commercial buildings such as hospitals, malls, restaurants which are beyond the scope of this review study. To search conference papers and reports, these keywords were searched only in the title. After the preliminary search, phase 2 included a three-stage literature filtration process. In the first step of the literature filter (filtration step 1), elimination was performed after careful reading of the abstracts, based on the inclusion criteria. This phase resulted in 84 studies. Next, we screened the full texts of the remaining 84 studies thoroughly, which resulted in 69 studies for further review. Finally, duplicate articles were eliminated, resulting in 61 papers for final review. The selected studies were classified into specific categories according to the aim of the review. Figure 2 shows the literature search overview with the selection criteria.

## 3. IAQ Standards & Assessment Methods

The quality of indoor air is crucial because people spend a significant portion of their time in different indoor spaces and also because of the presence of numerous pollution sources in indoor spaces, such as traditional and newly developed building materials, finishing products, furniture, cooking systems, and cleaning agents. Therefore, several international organizations worldwide, such as the WHO, have set guidelines and threshold values to maintain an optimal IAQ (Table 2). Apart from the WHO, the most recognized organizations involved in IAQ regulations include the American Society of Heating, Refrigerating, and Air-Conditioning Engineers (ASHRAE), US EPA, National Health and Medical Research Council in Australia, Health Canada, State Environment Protection Agency in China, Hong Kong Indoor Air Quality Objectives, Danish Society of Indoor Climate, Finnish Society of Indoor Air Quality and Climate, and Singapore Indoor Air Quality Guidelines [33].

Health problems due to IAQ, which are more commonly respiratory-related diseases and allergies, have increased the importance of IAQ measuring techniques and associated tools. Therefore, device types and monitoring systems of different indoor air pollutants were extensively reviewed. Table 3 shows a summarized list of identified indoor pollutants and devices used for pollutant detection.

## 4. Residential Buildings and IAQ Assessment

Realizing the potential risk of indoor air pollution, the Hong Kong government began establishing IAQ objectives in the last two decades. To fulfill this target, research has been performed to investigate the IAQ of local residential flats in Hong Kong [49]. Six housing were selected based on some pre-criteria, including three public rental houses and rest are private housing. Pre-criteria was developed to select those six homes, which includes housing type, highly populated location and finally housing not containing any newly purchased furniture during the sampling of indoor air. All the selected homes were occupied during the experiment, and both the kitchen and living room air was collected for investigation. The results of the investigation indicated that compared to the living room, the concentration of CO_2_ and PM_10_ were 14% and 67% higher in the kitchen, respectively. Similarly, the count of mean total bacteria was also 23% higher in the kitchen. Insufficient ventilation was indicated as the major reason for elevated CO_2_ level in the kitchen, whereas the impact of outdoor air infiltration, infrequent housekeeping, and mode of cleaning were found to have significant relation with higher PM_10_ level. The influence of cooking using liquefied natural gas was considerably higher in the case of VOC release when compared to natural gas-based cooking. Both new and established dwellings were assessed in Melbourne, Australia, to determine the VOC and HCHO levels [60]. Although the results indicated lower concentrations of VOCs in established homes; however, compared to the outdoors, these concentrations were four times higher. Moreover, attached garages, faulty wool carpeting, and site contamination were highlighted as sites of higher VOC concentration inside homes. New or renovated buildings yielded one- or two-orders higher VOC emission as compared to established buildings.

A study was conducted in Singapore to determine the relationship between the air quality inside bedrooms and sick building syndrome (SBS) for both naturally ventilated (NV) and air-conditioned (AC) systems [61]. The measured CO_2_ levels in NV bedrooms were lower than that in AC bedrooms. However, an opposite trend was observed in the case of particulate levels because NV bedrooms were found to have higher particulate levels. Another part of the assessment revealed higher SBS symptoms for residential occupants sleeping in AC bedrooms. As a part of the periodic assessment of building rules and regulations in England and Wales, several air quality parameters were examined in 37 homes [51]. This detailed study indicated gas cooking systems, occupancy, and location of the house as the three major contributors to a high level of inorganic gas emission. In accordance with the Canadian Environmental Protection Act (CEPA), a study was performed that investigated residential homes in Ottawa, Canada [58]. Compared to outdoor air, all targeted VOCs were present at a significant level in indoor air; however, their values were lower than that in the results of a study conducted in the previous year.

To determine the relationship between indoor PM_10_ and oxidative damage to plasmid DNA, a study was performed in China [62]. Houses with smokers and those with non-smokers were selected for air sampling. An investigation employing different technologies revealed that the PM_10_ generated in the living rooms and kitchens of smokers was more toxic and could cause 50% plasmid DNA damage, whereas the homes of non-smokers contained lesser bio-reactive PM_10_. The investigation determined that soot and unknown fine particles were responsible for plasmid DNA damage. To collect data on indoor pollutants, the Observatory on Indoor Air Quality in France examined a total of 567 dwellings and focused on over 30 different pollutants; they published two separate research studies [46,63]. The major VOCs found in these dwellings were formaldehyde, toluene, acetaldehyde, m/p-xylenes, and hexaldehyde. Simultaneous measurement of 20 VOCs in both indoor and outdoor spaces revealed that for a majority of the compounds, the median levels were considerably higher than those in outdoor spaces. Later in 2017, assessment of the same sampling size (567 dwellings) in mainland France was conducted to observe the relationship between measured air pollutants concentration and perceived IAQ [64]. In 2018, another study was conducted by OQAI (mandated by the French government) to assess the IAQ in energy-efficient buildings, which included both newly built and retrofitted buildings, based on standard quantification methods and questionnaires [47]. These energy-efficient buildings have been defined as consuming 40–75 kWh/m^2^ of energy per year in the case of newly built housing and 64–120 kWh/m^2^ per year in the case of retrofitted buildings. An analysis of the experimental data revealed higher concentrations of hexaldehyde, alpha-pinene, and limonene as compared to those from the abovementioned French studies. IAQ assessment in selected urban slams in Delhi, India for all three seasons (summer, rainy season, and winter) revealed 10 times higher air pollutants concentration during the winter period compared to the permissible limit [65]. Household characteristics such as occupants age, family size, types of kitchen and fuel, window opening facilities have been described in this study. However, the information related to building structures and arrangements (e.g., flooring type, furniture used) were not mentioned.

Despite the progress of the Korean government in the IAQ sector, a consumer agency survey reported that 14.5% of participants experienced SBS. Thus, a study was conducted to investigate the effects of several environmental factors on the IAQ of newly built apartments before and after occupancy, including construction characteristics, temperature, humidity, and occupation duration [54]. This investigation concluded that the average pollutant levels were in accordance with the guidelines set by the Ministry of Environment, except for the levels of formaldehyde and toluene. The study also attempted to correlate the pollutant behavior with temperature and humidity and strongly suggested evaluating the IAQ based on the load ratio of major pollutant sources. Finally, the study observed a reduction in the pollutant levels to approximately half of the initial values after one year of occupancy. A comparative study on aromatic VOCs was performed for residential houses in China and Japan during the period from 2006 to 2007 [66]. Smoking-related investigations were not the focus of this study, which was a limitation because smoking is a major source of indoor VOCs. The concentrations of investigated indoor VOCs were considerably higher in China as compared to those in Japan and the outside VOCs, indicating higher indoor VOC concentrations than those outdoors in the case of China; however, the Japan case study yielded consistent results for both indoor and outdoor VOCs. The carcinogenic analysis conducted in this study revealed an alarming 10 times higher exposure risk in China as compared to that in Japan. IAQ assessment in highly populated and polluted Lodi Province, Italy was performed for both gaseous pollutants and particulate matters [67]. Investigation in both summer and winter seasons concluded crossing the threshold value set by WHO for PM and NO_2_ for some cases, where CO and O_3_ level were found satisfactory. Although, reduction of pollutants produced through indoor sources was highlighted. However, no clear information about household characteristics was provided. Indoor Air Pollution and Health (IAPAH) study in Ireland and Scotland focused on IAQ assessment in homes with open combustion source [68]. Households with four different heating fuel were assessed including peat, coal, wood, and cooking gas, along with households with no open combustion source but having smoker occupants. Analysis of the air parameters concluded satisfactory level of pollutants in the households using gas stoves or solid fuels according to the WHO guideline values. However, poor IAQ occurred in the households with cigarette smokers. To characterize the patterns of airborne VOCs, an extensive study was performed in Leipzig, Germany; this study measured 60 different VOCs using a survey [56]. They used two different methods to allocate VOC compounds to their source of origin. An analysis of the enormous amount of sampling data concluded that occupant behavior, furnishing materials, ventilation, natural activities, and/or a combination of these factors significantly influence IAQ. To study the IAQ in harsh desert climates, an evaluation of gaseous and particulate matters was performed in Emirati houses in the UAE [69]. An attached garage (˂5 m from the house), the kitchen, and central AC systems were found to be mainly responsible for indoor PM_2.5_ and PM_10_, whereas an attached kitchen, smoking, and split AC were found to be significantly correlated with indoor CO levels.

The IAQ in a Leadership in Energy & Environmental Design (LEED)-certified green building in the US was assessed on an annual basis to ensure safe limits of indoor air pollutants [70]. Although green building regulations include certain steps, such as more supply of fresh air to the indoors and a selection of safe materials, to address the IAQ during the design phase, limited experimental data confirming the improvement of IAQ during the operation phase is available. The assessment revealed several benefits of the air quality in green buildings as compared to conventional buildings. According to the ASHRAE regulation, CO_2_ levels and relative humidity measurement data were at satisfactory levels. However, this study suggested that additional sensitive assessment techniques were required to more accurately assess the IAQ in green buildings. Impact of green renovation on IAQ was assessed in low-income housing apartment in Arizona, US before and after the retrofitting. Simultaneous air sampling (3 times: before, immediately after and 1 year later the renovation) and questionnaire survey concluded higher initial formaldehyde level which however met international standards after 1 year of retrofitting, except for 4% units [71]. 32% sampled houses in Macedonia were found to exceed the recommended limit of TVOC where the mean TVOC values were found to have a range from 50–2610 µg/m^3^ [6]. Analysis of the measured data in Ireland suggested that buildings exceeding the annual average concentration of 100 Bq/m^3^ should be further investigated in terms of their ventilation systems and current operations [59]. The small amount of sample data has been listed as a limitation of this study. The overall assessment concluded that passive houses mostly correspond with the threshold radon limit and perform better compared to conventional houses.

**Figure 3:**
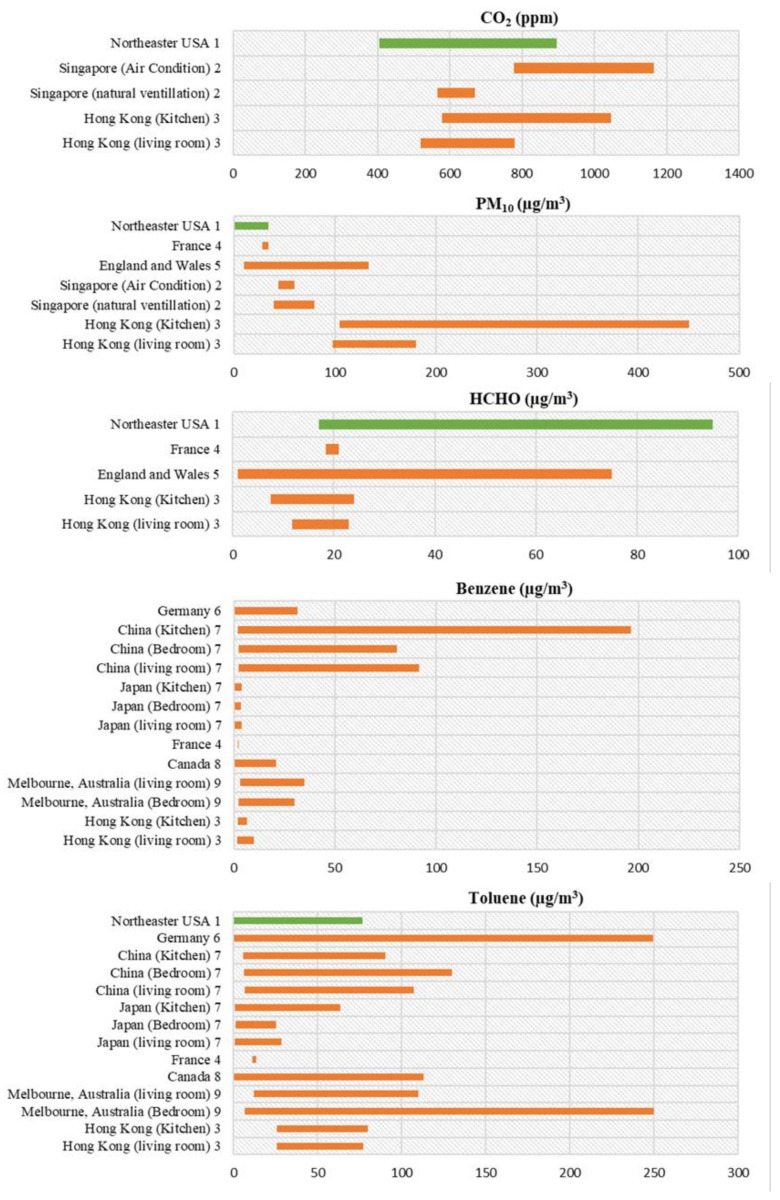
Range for five different indoor air pollutants concentration in residential buildings for selected countries. The numbers with each country’s name refer to the references. The indoor air pollutants concentration data for residential buildings collected for different countries from the following references: 1: [70]; 2. [61]; 3. [49]; 4. [46,63]; 5. [50]; 6. [56]; 7. [66]; 8. [58]; 9. [60]. The green color bar represents study results for green building.

Both quantitative and qualitative investigations of IAQ in rural communities (low-income families) were conducted to assess the impact of cooking fuels on PM_2.5_ and CO concentration in Paraguay [72]. A much higher concentration of PM_2.5_ and CO were found in houses using charcoal and wood compared to the houses using electricity and LPG for cooking, exceeding the recommended value by WHO. Impact of building retrofitting activity on IAQ was assessed in multifamily housing in Lithuania and Finland [73]. Significant reduction in both fungal and bacterial concentration was found after retrofitting activity in Finnish housing, where the opposite trend was found for selected VOCs. Investigation in Lithuanian housing concluded significant increase in radon concentration as a result of retrofit. To assess the IAQ in both new and renovated housing with gas cooking burners, a study was performed in California where the resident density was higher [74]. This comparative study indicated 165% increase in NO_2_, 18% increase in CO_2_, 25% decrease in formaldehyde, and 4% decrease in PM_2.5_ when compared to a recent study performed in code-compliant ventilated California buildings. The later study was performed in 70 detached buildings in California and resulted in a significant decrease in formaldehyde and PM_2.5_ compared to the study conducted for the new homes in California for the period 2006–2007 [75]. Although having limitations, these results are invaluable for the improved IAQ in future retrofit housing. Figure 3 represents the range for five different indoor air pollutants concentration in residential buildings for selected countries discussed above, while Table 4 has summarized different aspects of residential IAQ studies.

## 5. Commercial Buildings and IAQ Assessment

The IAQ in schools has been one of the major concerns among researchers, because children are more susceptible to air pollutants than adults, and children spend a significant amount of time in schools. Similarly, the IAQ in office buildings has also been of particular concern because it significantly affects the productivity of workers. Realizing the research gap in this field, in 2003, a large number of commercial buildings (20 offices, 4 schools, 1 hospital, and 1 nursing home), as well as dwellings and building products, were assessed to investigate the presence of indoor VOCs in Australia [76]. A total of 163 VOCs were identified in indoor environments as well as during the product analysis process, and a majority of the observed VOCs were released from different indoor building materials and a few surface finishes and appliances. The Korean government has also expended significant effort for improving educational conditions; as a part of these efforts, a few studies have been performed to characterize IAQ. To assess the severity of indoor air pollutants based on the age of school buildings, a study focusing on 55 different schools was conducted in Korea [51]. The factors that contributed to indoor air pollution in schools were listed as emissions of chemicals from building materials or furnishings and unsatisfactory ventilation. The HCHO concentration was found to be significantly higher than the standard value established by the Korean government. Considering the higher susceptibility of younger kids as compared to higher grade children, another study focusing on preschools was conducted in Korea [55]. The study concluded that preschools in urban areas have considerably higher indoor pollution levels as compared to the outside and rural preschool environments in Korea. In naturally ventilated office space inspection, influencing factors for indoor particulate matters were indicated as the nearby construction activity, indoor human movement, tobacco smoke, and computer operation [77]. Investigation of school IAQ in West Macedonia, Greece highlighted the presence of many times higher PM_10_ concentration compared to the outdoor in normal operation period [78]. To determine the correlation between indoor pollutant levels in several primary schools, a study was conducted in Belgium [79]. This study investigated 7–8-year-old school kids for exposure to NO_2_, SO_2_, O_3_, BTEX (benzene, toluene, ethylbenzene, xylene isomers), and PM_2.5_. Higher benzene concentrations were observed in lower level classrooms, and the carpet in classrooms was determined to be responsible for the higher PM_2.5_ concentrations.

The impact of airborne fungi on the IAQ inside AC offices was assessed in compliance with the new IAQ policy of 2003 in Hong Kong [80]. The assessment yielded satisfactory results that indicated a decrease in the count of airborne fungi after applying the new IAQ policy, except for the CO and NO_2_ emission rates. To investigate ammonia (NH_3_) contamination in an office located in Beijing, a study combining standard experiments and a questionnaire was conducted [42]. The results of this study were compared to the results gathered from the office of the same company located in Stockholm. This comparative study revealed a significantly higher concentration of NH_3_ (3–6 ppm) in the Beijing office, whereas its concentration was ˂0.1 ppm for the office in Stockholm. Similarly, a considerably higher benzene concentration was observed in Beijing; the concentrations were 26.8 and 0.4 µg/m^3^ for Beijing and Stockholm, respectively. This study indicated that a concrete additive was the probable source of NH_3_ emission.

A study performed in Michigan State, USA for nine elementary and middle schools resulted in satisfactory bioaerosol and VOC levels [44]. The possible sources of VOCs were listed as a combination of indoor and outdoor sources along with occupant behavior, while carpets were identified as a possible source of bioaerosols. In total, 37 semi-randomized small and medium commercial buildings (e.g., offices, restaurants) were investigated in California, USA to monitor the particle concentrations [81]. Continuous measurement of the sample buildings resulted in an indoor–outdoor particulate matter ratio of less than one for the majority of the buildings. This study also indicated the disadvantage of low-efficiency filters in most of the observed buildings, which allowed outdoor particles to enter inside. Apart from the building materials and other processes inside buildings, the role of humans in IAQ was assessed based on a university classroom in Boulder, Colorado, USA by a continuous VOC measurement process [57]. The VOC measurement analysis confirmed that respiratory emissions from human beings and reaction of O_3_ with their skin lipids affects the indoor VOC concentration. These human activities were found to be responsible for 40% of the daytime VOC. To investigate the IAQ for a range of climates in the US, another research group performed a simulation study using the building energy model “EnergyPlus,” and it was found that PM_2.5_ was mostly affected by changing weather patterns and ventilation systems, where CO_2_, HCHO, NO_3_, and O_3_ concentrations were independent of ventilation patterns [82].

Particulate matters (PM) are considered as critical pollutants in most parts of India; this was determined while investigating a school in Chennai, India [45]. The significant concentrations of PMs were found to exceed the National Ambient Air Quality Standards in India. Rather than indoor pollution sources, outdoor sources, including ambient particles emitted by traffic, were found to be responsible for these concentrations. Another study focused on non-commercial buildings (offices and educational institutes) and IAQ was conducted in Delhi, India [52]. This study highlighted the significant impact of occupant density on indoor air pollution in the case of non-residential buildings. The CO_2_ concentrations in two office buildings were significantly higher than the ASHRAE baseline; moreover, the measured concentrations of pollutants in educational buildings were lower than those in office buildings. Ductless air conditioning systems and ineffective air circulation systems were the primary contributors to the higher PM_2.5_ in the office buildings. A study conducted in two states of the UAE revealed the poor IAQ conditions in 16 elementary grade classrooms [83]. The measured concentrations of TVOC, CO_2_, and particles were 815 µg/m^3^, 1605 ppm, and 1730 µg/m^3^, respectively, whereas the recommended values for these concentrations established by the Dubai Municipality were 300 µg/m^3^, 800 ppm, and 150–300 µg/m^3^, respectively.

To understand the influence of children’s activity on IAQ, an evaluation was performed in nursery schools located in Poland [84]. Investigated indoor pollutant concentrations were found to be higher compared to the outside ones, such as PM_2.5_ and PM_10_ that were in the ranges of 41.17–106.06 µg/m^3^ and 68.26–149.81 µg/m^3^, respectively, exceeding WHO indoor guidelines. The effect of increased ventilation on classroom IAQ was investigated in 18 classrooms in the Netherlands [85]. Results indicated a much lower concentration of endotoxin, b(1,3)-glucan, and PM_10_ because of increased ventilation, whereas there was no such effect on PM_2.5_ and NO_2_ levels. However, analysis of Italian classrooms concluded that greater manual airing resulted in higher sub-micrometric particulate penetrations in indoor classroom environments [43]. An investigation of indoor air in office environments focused on PM_2.5_ and PM_10_ on both normal and dusty days over a 2-month period in Qatar [86]. The study concluded that significant concentrations of PMs in indoor spaces are mainly caused by ventilation, faulty building envelopes, and windows. In comparison with the ASHRAE and US EPA IAQ standards, concentration of CO_2_ and particulate matters were much higher during a study of Qatari schools, and outdoor PM levels were identified as the main reason for high indoor particulate matter levels [87]. A study of Turkish university classrooms concluded higher CO_2_ and PM levels and observed a radon concentration that was lower than the standard value set by the International Commission on Radiological Protection, but it was higher than other worldwide values [88]. To increase the knowledge about IAQ in modern offices, a project was performed in Europe that focused on newly built and refurbished office buildings in Europe [53]. All the indoor pollutants, except xylene, showed significant variations for different seasons (summer and winter). The association between human health and IAQ assessment indicated a higher PM_2.5_ concentration, whereas formaldehyde, ozone, acrolein, α-pinene, and D-limonene concentrations were in acceptable ranges. This study recommended to perform a pollution assessment in at least two different time periods during a year along with an assessment of both ground and highest floors. Under the same project, another study showed a correlation between aldehyde, VOC levels, and factors associated with building structures and resident behavior [89].

Recently, new low-energy regulations for new buildings in the European Union stipulated that all new schools built in this region should follow airtight and energy-efficient envelope. To investigate the IAQ of these newly built low-energy preschools in Sweden, a comparative study was performed [48]. A strong relationship was found between IAQ and the functioning level of the ventilation system. The comparative study concluded that the preschool manufactured with Swan eco-labeled materials emitted fewer initial TVOCs compared to preschools made with conventional materials. In compliance with this trend, seven low-energy schools in Sweden were also investigated, and analysis of the data confirmed a satisfactory temperature and CO_2_ level as set by the Swedish guidelines [90]. Investigation of IAQ in schools located in a highly polluted area of southern Italy was performed, which resulted in higher CO_2_, NO_2_, PM _2.5_, and endotoxin levels, whereas the Der p 1 allergen level was found to be below the threshold limit [91]. Table 5 has summarized the IAQ studies in different locations.

## 6. Conclusions and Future Scope

This paper, which discusses the last 20 years of indoor air research, aimed to review the IAQ sector from different aspects to understand the interaction between IAQ and building environments, which were mainly residential and a few commercial buildings. Since people spend over 85–90% of their time in different buildings, the IAQ of different types of building can have a major impact on human health. Therefore, this paper has done a review on the current state of the art and knowledge related to the IAQ of different residential and commercial buildings. Moreover, to understand the progress in IAQ research around the world, studies from different countries were reviewed. Region/location-specific review can also be beneficial to identify the major indoor air pollutant in each location, which need to be addressed for long term solutions. Hence, this review aimed to benefit building professionals when creating new indoor air regulations, considering major air pollutants, all indoor contaminant sources, and related health impacts, to create healthy and sustainable building environments.

Most of the developed countries consider and follow IAQ regulations during the design and maintenance phase of building environments through appropriate measures. However, this scenario is not similar in developing or underdeveloped countries, where poor IAQ disproportionately affects children, women, and elderly persons [92]. Despite the severe impact of exposure to indoor air pollutants, there is still a lack of proper scientific research on IAQ in most developing and underdeveloped countries/regions. Analysis of peer-reviewed journals during this review indicated that primarily developed and a few developing countries are more interested in exploring IAQ in terms of the human health impact, whereas underdeveloped countries still lack IAQ-focused research. The pattern of indoor air pollutants in developing and underdeveloped countries and the consequences to health should be studied more, which can provide a baseline to determine more beneficial IAQ policies in these regions. Therefore, more research is needed in these regions to ensure healthy and sustainable building environments worldwide. Along with indoor pollution sources, the situation of IAQ is worse in some regions because of outdoor climatic conditions, such as high humidity, temperature, and dust intensity, such as in GCC countries. However, studies that have focused on the IAQ situation in GCC countries have mostly excluded detailed VOC evaluations.

The reviewed studies commonly examined some parameters, such as PM, volatile matters, carbon dioxide, and carbon monoxide; however, most have focused on selected VOCs. Although a few studies have analyzed VOCs in detail, most limited their studies to estimating TVOC, benzene, toluene, xylene, and ethylbenzene. Most studies have preferred to use gas chromatography-mass spectrometry to analyze VOCs, showing that it is the most popular detection method for VOCs. Among the reviewed studies, analysis of carcinogenic air pollutants, such as radon, was rare. Additionally, few studies have clearly reported the building materials in walls or floors, whereas others did not mention the finishing type, furniture material, cleaning agent, household activities, which are highly critical elements for analyzing IAQ. Similarly, most studies focusing on commercial building’s IAQ have not specified the specific detail of the indoor materials that has the most impact on the air pollution. However, building structure and/or materials, surface finishes, and resident’s activity in general have been indicated as the major reasons for the elevated VOC concentration in the reviewed commercial buildings. Similarly, outside PM level and/or nearby construction process, tobacco smoke, presence of carpet, human movement have been identified for rise in indoor PM level where concrete additives has been indicated as the responsible element for higher indoor NH_3_ concentration. Moreover, inter-relation model or equation between pollutants concentration and pollution source inside building environment was not clearly presented in the reviewed studies. Therefore, this study recommends more studies focusing on detailed assessment of exposure concentration along with the identification of responsible sources in each type of building environment.

Of note, direct comparison of indoor air pollutant levels is difficult and not straightforward because evaluations have been conducted over different time periods, using different instruments and sampling techniques, and in different indoor environments. Thus, it is highly recommended that more detailed scientific studies be conducted by following standardized regulations, which will allow for an inter-comparison of IAQ from studies in the future to close the existing knowledge gaps regarding IAQ.

## Figures and Tables

**Table 1:** Indoor air contaminants list and related health impacts.

Contaminants	Sources	Possible Consequences	Ref.
**Biological Contaminants**			
Allergens	Furry pets, dust mites	Asthma	[10,11]
Endotoxins	Presence of cats and dogs, contaminated humidifiers, storage of food waste, lower ventilation rate, increased amount of settled dust	Asthma, reduced lung function	[12,13]
Dampness and mold	Unattended plumbing leaks, leaks in building fabric, hidden food spills, standing water	Upper respiratory symptoms, cough, wheeze, and asthma	[14,15]
**Chemical Contaminants**			
Smoke	Tobacco smoke	Premature mortality, lung cancer, coronary artery disease, childhood cough and wheeze, respiratory illness, infant death syndrome	[16,17]
Coal & biomass fuels combustion product	Cooking and heating	Combustion of solid fuels releases CO, N_2_O, particulates, poly-cyclic hydrocarbons, which increases risk of lung cancer, childhood asthma	[18,19]
Carbon Monoxide (CO)	Vehicle exhaust from attached garages, gas stoves, furnaces, woodstoves, fireplaces & cigarettes	Headache, nausea, fatigue	[20,21]
Nitrogen dioxide (N_2_O)	Combustion of fossil fuels e.g., gas or oil furnaces and stoves	Increased risk of respiratory symptoms	[22,23]
Pesticides	Contaminated soil, stored pesticide containers	Irritation to eye, nose, and throat, damage to central nervous system	[24,25]
**Off Gassing Emissions (Gases released from indoor materials)**			
Formaldehyde (HCHO)	Wood-based products assembled using urea-formaldehyde resins, cigarette smoke, paints, varnishes, floor finishes	Eye, nose, throat irritation, asthma, bronchitis, and possible carcinogen	[26,27]
Volatile Organic Compounds (VOC)	Cigarette smoke, recently dry-cleaned cloths, room deodorizers, paints, carpets	Asthma, bronchial hyper-reactivity	[28,29,30]
Plastic Compounds	Polyvinyl chloride for flooring, plastic wall material	Bronchial obstruction, asthma, wheeze, cough, and phlegm	[31]
**Carcinogens**			
Radon	Natural decay of uranium	Lung cancer, leukemia	[26,32]
**Particulate Matter**			
Ultra-fine particles	Cooking, combustion activities	Serious impact on heart and lungs	[7,26]

**Table 2:** Standards for indoor air quality (IAQ) by international organizations/Government.

Parameters	CAS	WHO [34]	Singapore [35]	NIOSH [36]	Canada [37]	China [38]	UK [39]	Australia [40]	US EPA [41]
Benzene (C_6_H_6_)	71-43-2	No safe level of exposure can be recommended	-	-	-	90 ug/m^3^ [1 h avg.]	-	-	-
Carbon Di-oxide (CO_2_)	124-38-9	100 mg/m^3^ (15 min) 35 mg/m^3^ (1 h) 10 mg/m^3^ (8 h) 7 mg/m^3^ (24 h)	1000 ppm (8 h avg.)	5000 ppm (8 h avg) 30,000 ppm (15 min)	≤6300 mg/m^3^ (≤3500 ppm)	1000 ppm (daily avg.)	15,000 ppm (15 min avg.) 5000 ppm (5 min avg.)	30,000 ppm (15 min avg.)	800 ppm
Carbon mono-oxide (CO)	630-08-0	86 ppm (15 min avg.) 51 ppm (30 min avg.) 25 ppm (1-h avg.) 8.6 ppm (8-h avg.)	10 mg/m^3^ (9 ppm) (8 h avg.)	35 ppm (8 h avg.)	≤11 ppm (8 h avg) ≤25 ppm (1 h avg.)	5.0 mg/m^3^ (daily avg.)	11.6 mg/m^3^ (8 h avg.)	9 ppm (10,000 μg/m^3^) (8 h avg.)	35 ppm (1 h) 9 ppm (8 h)
Formaldehyde	50-00-0	mg/m^3^ (30 min) 0.2 mg/m^3^ (long term)	0.1 ppm (120 μg/m^3^) (8 h avg.)	0.016 ppm 0.1 ppm (15 min)	120 µg/m^3^	0.12 mg/m^3^ (1 h avg.)	2 ppm (15 min avg.) (2500 μg/m^3^)	2500 μg/m^3^ (15 min avg.)	920 μg/m^3^ (8 h)
Naphthalene	91-20-3	0.01 mg/m^3^ (annual avg.)	-	-	-	-	-	-	-
Nitrogen dioxide	10102-44-0	200 μg/m^3^ (1 h) 40 μg/m^3^ (annual avg.)	-	1 ppm (15 min)	≤100 µg/m^3^ ≤480 µg/m^3^ (1 h)	0.10 mg/m^3^ (daily avg.)	200 μg/m^3^ (1 h) 40 μg/m^3^ (1 year)	-	0.053 ppm
Polycyclic aromatic hydrocarbons	83-32-9	No threshold can be determined	-	-	-	-	-	-	-
Trichloroethylene	79-01-6	4.3 × 10^−7^ μg/m^3^ (unit risk)	-	-	-	-	-	-	-
Tetrachloroethylene	127-18-4	0.25 mg/m^3^ (annual avg.)	-	-	-	-	-	-	-
Ozone	10028-15-6	-	0.05 ppm (8 h avg.) (0.100 mg/m^3^)	0.1 ppm	≤240 µg/m^3^ (1 h)	0.1 mg/m^3^ (1 h avg.)	100 μg/m^3^ (8 h)	0.1 ppm (1 h) 0.08 ppm (4 h)	0.12 ppm (1 h) 0.08 ppm (8 h)
Sulfur dioxide (SO_2_)	7446-09-5	-	-	2 ppm (8 h avg.) 5 ppm (15 min)	≤50 µg/m^3^ ≤1000 µg/m^3^ (5 min)	0.15 mg/m^3^ (daily avg.)	-	0.25 ppm (10 min) 0.2 ppm (1 h)	0.5 ppm (3 h) 0.14 ppm (24 h) 0.03 ppm (1 year)
Relative Humidity (RH)	-	-	<70%	-	30–80%—summer; 30–55%—winter	-	-	-	-
Radon (Rn)	10043-92-2	-	-	-	800 Bq/m^3^ (1 yr avg.)	-	-	-	-
PM_2.5_	-	25 μg/m^3^ (24 h avg.) 10 μg/m^3^ (annual avg.)	-	-	≤40 µg/m^3^ ≤100 µg/m (1 h)	-	-	-	65 μg/m^3^ (24 h)
PM_10_	-	50 μg/m^3^ (24 h) 20 μg/m^3^ (1 year)	150 μg/m^3^ (in office)	-	-	0.15 mg/m^3^ (24 h)	-	90 μg/m^3^ (1 h avg.)	150 μg/m^3^ (24 h) 50 μg/m^3^ (1 year)

**Table 3:** Common IAQ measurement techniques.

Sampling Item	Sampling Methods/Tools	Sampling Duration/Cautions	Ref.
CO_2_, RH, temperature	Q-Trak monitor (TSI Inc.): Nondispersive infrared analyzer	Sampling duration: 7 days, 10 min (min) average	[30,35,37,42,43]
	Integrated data loggers (Hobo HO-8)	Sampling in every 5 min	[44]
	Indoor air quality meter (IAQ-CALC model 7545)	NA	[45]
CO	Electrochemical sensor (Draeger Pac III) FIM CO- Tester Tx for exhaled air	Sampling duration: 7 days, 5 min average	[46]
NO_2_	Passive samplers (Palmes tubes) containing triethanolamine absorbent and analyzed by a spectrophotometer	NA	[47,48]
PM_10_	Dust-Trak air monitor (Model 8520, TSI Inc.), Light scattering	Sampling rate: 1.7 L/min, 1-min interval	[49]
	Pumped gravimetric method	Sampling duration: 24 h	[50]
	Model 2100 Mini- Partisol air sampler (Ruprecht & Patashnick Co.) coupled to a ChemPass model 3400	37 mm diameter membrane (2 µm porosity) was used to collect particulate matters	[46]
	GRIMM environmental dust monitor, light scattering technology	Sampling rate: 1.2 L/min, for 2 weeks (suitable for PM2.5 and PM1 also)	[45]
	Minivol portable air sampler (Airmetrics, PAS 201) with pall flex membrane filter (47 mm)	Filter conditioned in dry air for 48 h, sampling duration 5–7 h	[51]
PM_2.5_	PTFE filters (37-mm diameter, 2-μm porosity)	Sampling rate: 1.8 L/min using a personal impactor, duration: 5 p.m. to 8 a.m. on weekdays and 24 h on weekends. Passive samplers and PM filters were stored in a freezer to keep them cool and avoid sunlight exposure	[47]
	Low volume sampling pump (model 224-PCXR8) with PEM impactor	Every 5 min intervals	[52,53]
Airborne bacteria	Burkard single stage impactor (Burkard Manufacturing Co. Ltd.) with an agar plate, followed by colony counting	Sampling rate: 10 mL/min for 9 min, incubated at 35 °C in an oven for 2 days	[49]
HCHO	SKC formaldehyde monitoring kit: Colorimetric method	Sample should be refrigerated and protected from sunlight and immediately sent to the air laboratory for analysis within 1 h	[49]
	Sample collection: Portable pump (Flec-FL. 1001 or Sibata) with 2,4-DNPH cartridge connected with ozone scrubber. Analysis: two stage thermo desorption followed by gas chromatography/mass spectroscopy	30 min ventilation of housing unit followed by 5 h of sealing. Samples were taken after that, 30 min each.	[54]
	Radial diffusive samplers filled with 2,4-dinitrophenylhydrazine (2,4-DNPH)-coated Florisil (Radiello^®^ code 165), analyzed by liquid chromatography with detection by UV absorption	Sampling duration: 2 weeks	[47,48]
	Diffusion sampler SKC UMEx100 based on chemosorbtion on 2,4-dintrophenyl htydrazine, analyzed by liquid chromatography	Sampling duration: 1 week	[42]
	Air pull through 2,4-dinitrohydrazine (DNPH) coated silica gel cartridge (Supeleo LPDNPH S10)	Sampling rate: 0.2 L/min for 40 min	[51,55]
VOC	Mass flow controllers (Model No. FC4104CV-G, Autoflow lnc.) trapped by Nutech Cryogenic Concentrator (Model 3550A), analyzed by Hewlett Packard Gas Chromatography (GC) (Model HP6890) using TO-14 method	Sampling rate: 0.011 L/min for 8-h	[49]
	Diffusive samplers	Exposure period of three days to two weeks	[50]
	Radial diffusive sampling onto carbograph 4 adsorbents (Radiello^®^ code 145), analyzed by gas chromatography-mass spectrometry	Sampling duration: 7 days	[46,47]
	Passive sampling (diffusion principle) with organic vapor monitors	Middle of the room, height: 1.5 to 2 m	[56]
	Thermal desorption tube, analyzed by gas chromatograph/mass selective detector (GC/MSD)	Sampling rate: 0.07∼0.1 L/min	[44,55]
	Proton transfer reaction mass spectrometer (PTR-MS)	Sampling duration: Less than 5 min	[57]
	Tenax-TA tubes, analyzed by gas-chromatography with flame ionization detection (Varian, model 3700) & modified thermal desorption	Sampling rate: 20 mL/min for 40 min	[48,51]
	Air pumped through a charcoal filter (Anasorb 747)	Sampling rate: 250 mL/min for 4 h	[42]
	Air collected on adsorbent tubes and analyzed by gas chromatography-mass spectrometry	Sampling rate: 100 mL/min for 100 min	[58]
	Organic vapor sampler, adsorbed on activated charcoal column, analyzed by gas chromatography-mass spectrometry	Sampling duration: 8 h	[45]
TBC	RCS sampler (Biotest air samplers) following centrifugal impaction principle	Sampling rate: 40 L/min for 4 min	[51]
Rn	CR-393 alpha track diffusion radon gas detectors	Sampling duration: 3 months	[59]
	Alpha Guard Professional Radon Monitor	Sampling duration: 1 week	[43]
	Passive measurements of Radon volumic activity by accumulating alpha radiation on 12 m cellulose nitrate film (Kodalpha dosimeter)	Sampling duration: 2 months	[46]
	Passive dosimeters (Kodalpha LR 115 detectors)	Sampling duration: 2 months, only in heating season	[47]
Gamma	Gamma radiometer of the Geiger-Muller type (Saphymo 6150 AD6)	Sampling duration: 3–4 h	[46]
Total Suspended Particulates & respirable suspended particulates (TSPs & RSPs)	PVC filters (pore size 0.45 μm, diameter 37 mm, SKC, USA)	Sampling rate: 2.5 L/min	[55]
Lead (Pb)	Airborne lead: mixed cellulose ester filter (pore size 0.8 μm, diameter 37 mm), analyzed with a Varian GTA100 model graphite furnace mounted on a Varian SpectrAA-880 model atomic absorption spectrophotometer based on NIOSH method 7105 Surface lead: collected with wet tissues based on NIOSH method 9100	Sampling rate: 4 L/min	[55]
Ammonia (NH_3_)	Kitagawa precision gas detector tubes	NA	[42]
Airborne asbestos	Open-faced mixed cellulose ester filter (37 mm diameter and 0.8 μm pore size)	Sampling rate: 2.5 L/min	[55]
Airborne micro-organism	25 mm nucleopore filter	Pore size 0.4 nm, sampling rate 2 L/min for 4 h	[42]
Mold & bacteria	CAMNEA method	Sampling rate: 4 h outside the window	[42]
Bacterial aerosols	Swirling liquid impingers	Sampling rate: 12.5 L/min	[45]

**Table 4:** Summary of the residential IAQ research in different locations.

Investigation Location	Sample Number	Study Area	Indoor Material	Ventilation	Parameters Examined
Hong Kong (2002), [49]	6	Living room, Kitchen	Plastering wall, wallpaper, tile/wood/vinyl floor	Natural ventilation with air conditioning	CO_2_, HCHO, PM_10_, Bacteria, C_6_H_6_, C_6_H_5_CH_3_, C_6_H_5_CH_2_CH_3_, C_6_H_5_(CH_3_)_3_, CHCl_3_, CH_2_Cl_2_
Australia (2002), [60]	27 (ED) * & 4 (NB) *	Living room, bedroom	NA	NA	VOC, HCHO
Singapore (2004), [61]	3	Bedroom	NA	Natural ventilation with air conditioning	CO_2_, RH, particulate profile, bacteria, fungi, temperature
England & Wales (2005), [50]	37	Living room, kitchen, other rooms	timber framed construction, traditional brick/block frame, cavity wall insulation	mechanical extract ventilation and passive stack ventilators	NO_2_, CO, HCHO, VOC, RH particulates, temperature
Ottawa, Canada (2005), [58]	75	Living room and family room	NA	NA	37 VOCs
China (2007), [62]	6	Living room, Kitchen	NA	NA	PM_10_
France (2008), [46,63]	567	Rooms, attached or integrated garages and outside the dwellings	NA	NA	CO, VOC, particles, Rn, dog, cat and dust mite allergens, radon and gamma radiation
India (2008), [65]	5	Kitchen, bedroom	NA	Natural Ventilation	particulate matter (RSPM), CO_2_, CO, SO_2_, and NO_2_
Korea (2009), [54]	158	Living room, kitchen, master room, other room	Wall & ceiling: Silk/Balpo, floor: PVC/wood, furniture: MDF	NA	HCHO, VOC, C_6_H_6_, C_6_H_5_CH_3_, C_6_H_5_CH_2_CH_3_, (CH_3_)_2_C_6_H_4_, C_6_H_4_Cl_2_, C_6_H_5_CH=CH_2_
China & Japan (2009), [66]	57 (Jp) & 14 (Ch)	Living room, kitchen, bedroom	Wallpaper (Japan); paint (China)	NA	VOC (C_6_H_6_, C_6_H_5_CH_3_, C_6_H_5_CH_2_CH_3_, (CH_3_)_2_C_6_H_4_, C_6_H_5_(CH_3_)_3_
Italy (2011), [67]	60	Living room	NA	NA	PM, NO_2_, CO, O_3_
Ireland & Scotland (2011), [68]	100	Living room	NA	NA	PM_2.5_, CO, CO_2_, NO_2_
Germany (2013), [56]	2246	Living or child’s room	NA	NA	60 VOC’s
UAE (2014), [69]	628	Family room	NA	Sealed AC	CO, HCHO, H_2_S, NO_2_, SO_2_, PM_2.5_, PM_10_
United States (2015), [70]	17	NA	Hardwood floors, carpets	Natural ventilation with air conditioning	CO_2_, CO, RH, temperature, particulate matter, VOC, HCHO
United States (2015), [71]	86	Living room and kitchen	Low VOC carpet, flooring, carpet pad, zero VOC paint	HVAC system	PM, HCHO, VOC
France (2017), [64]	567	Bedroom and living room	NA	Mechanical ventilation	CO_2_, RH, VOCs, HCHO, PM_2.5_, PM_10_
France (2018), [47]	72	Living room, master bedroom	Lightweight/masonry facades, timber frame, thermal insulation	Mechanical or hybrid ventilation	CO_2_, CO, RH, NO_2_, VOCs, HCHO, Rn, airborne particles, temperature
Macedonia (2017), [6]	25	Living room	NA	NA	Temperature, RH, TVOC, PM
Northern Ireland (2019), [59]	5	Main living area, bedroom	Timber & Masonry	Balanced mechanical heat recovery ventilation or demand-controlled ventilation systems	Rn
Paraguay (2019), [72]	80	Kitchen	NA	NA	PM_2.5_, CO
Finland & Lithuania (2019), [73]	45	Living room	NA	Natural and mechanical ventilation	CO, NO_2_, VOCs, Rn, microbial content
California, USA (2020), [74]	23	Bedroom, living room, kitchen, dinning area	NA	Mechanical ventilation	CO_2_, NO_2_, HCHO, PM_2.5_
California, USA (2020), [75]	70	Bedroom, living room	NA	Mechanical ventilation	CO_2_, NO_2_, HCHO, PM_2.5_, NOx, RH, temperature

Notes: NA = Not available/applicable, ED = Established dwellings, NB = New buildings, C_6_H_5_CH_3_ = Toluene, C_6_H_5_CH_2_CH_3_ = Ethylbenzene, C_6_H_5_(CH_3_)_3_ = Trimethylbenzene, CHCl_3_ = Chloroform, CH_2_Cl_2_ = Methylene chloride, (CH_3_)_2_C_6_H_4_ = Xylene, C_6_H_4_Cl_2_ = 1,4-dichlorobenzene, C_6_H_5_CH=CH_2_ = Styrene, H_2_S = Hydrogen Sulfide.

**Table 5:** Summary of the commercial buildings IAQ research in different locations.

Investigation Location	Sample Number	Seasonal Variation	Indoor Material	Ventilation	Parameters Examined
Australia (2003), [76]	20 office, 4 schools, 1 hospital & 1 old home	NA	NA	NA	VOC
Korea (2007), [51]	55 schools, 30 std/class	Summer, autumn, winter	Pressed wood desks, chairs, furnishings	Mainly naturally ventilated	CO, CO_2_, PM_10_, TBC, TVOCs, HCHO
Korea (2011), [55]	17 pre-schools (71 classrooms)	Late spring and summer	Concrete, floor covered with linoleum/wood, no carpet	Naturally ventilated	TSPs, RSPs, lead, asbestos, TVOCs, HCHO, and CO_2_
Greece (2007), [77]	3 (office)	Spring	glazed windows. Painted gypsum board wall, plastic tiles, no carpet	Natural ventilation	PM
Greece (2008), [78]	1 (school)	Summer, fall, and winter	NA	Natural ventilation	PM_10_, O_3_, CO
Antwerp, Belgium (2008), [79]	27 (primary school)	Winter and early summer	NA	Natural ventilation	PM_2.5_, K, Ca, Ti, V, Cr, Mn, Fe, Ni, Cu, Zn, Br, Pb, Al, Si, S, Cl, NO2, SO_2_, O_3_, and C_6_H_6_, C_6_H_5_CH_3_, C_6_H_5_CH_2_CH_3_, and (CH_3_)_2_C_6_H_4_
Hong Kong (2008), [80]	82 (office)	NA	NA	mechanically ventilated and air-conditioned	Airborne fungi count
Beijing (2009), [42]	2 (office)	Spring and early summer	NA	Mechanical ventilation	RH, HCHO, VOCs, NH_3_, CO_2_, mold and bacteria
Michigan, USA (2007), [44]	64 (school)	Spring and early summer	Carpet	Mechanical ventilation	Ventilation rates, VOCs and bioaerosols, CO_2_, RH, and temperature
California, USA (2012), [81]	37 (office & others)	NA	NA	Rooftop heating, ventilation, and air conditioning units	Black carbon, PM_2.5_, PM_2.5-10_, PM_10_
Colorado Boulder, USA (2016), [57]	1 (university)	Spring	Latex paint in wall	Dedicated air handling unit	VOC
USA (2016), [82]	14	All seasons	NA	2 Mechanical ventilation & 2 natural ventilation	CO, CO_2_, HCHO, NO_2_, O_3_, PM_2.5_
Chennai, India (2012), [45]	1 (school)	Winter & summer	NA	Natural ventilation	PM_10_, PM_2.5_, PM_1_, CO, HCHO, bioaerosols
Delhi, India (2017), [52]	3 (2 office & 1 EB*)	June-July	Concrete flooring	Air condition	CO_2_, PM_2.5_, VOC
Dubai & Fujairah, UAE (2014), [83]	16 (elementary school)	Summer & winter	NA	NA	TVOC, CO_2_, O_3_, CO, particle concentration
Gliwice, Poland (2015), [84]	2 (Nursery school)	Winter	NA	Stack ventilation and airing	VOC, PM, bacterial and fungal bioaerosol, CO_2_
Netherland, (2015), [85]	17 (Primary school)	Winter	NA	Naturally ventilated	Endotoxin, b(1,3)-glucans, PM_10_, PM_2.5_, NO_2_
Italy (2016), [43]	7 school (16 Classrooms)	Winter & spring	Single/double glazed Al/Fe window	Manual airing	CO_2_, particulate concentration, Rn
Qatar (2017), [86]	1 (Office Building)	Summer	NA	HVAC	PM_10_, PM_2.5_
Qatar (2017), [87]	16 (urban schools)	Winter	Floor: vinyl or ceramic tile	Mechanically ventilated	temperature, RH, CO, CO_2_ and particulate matters (PM_10_ and PM_2.5_)
Turkey (2018), [88]	4 (university classrooms)	Winter & summer	Desk & table: MDF veneered compressed chipboards, Door: woodwork	Natural ventilation	Temperature, RH, CO_2_, Rn, PM_0.5_, PM_1.0_, PM_2.5_, PM_5.0_, and PM_10_
Europe (2016), [53]	37 (office)	Winter & summer	NA	Mostly mechanical ventilation	VOC, HCHO, O_3_, NO_2_, PM_2.5_
Europe, (2019), [89]	37 office (140 office room)	Winter & summer	Synthetic floor covering, dispersion or emulsion wall paint, furniture: wood and derivatives (45%) or metal (31%), ceiling: synthetic	Mostly mechanical ventilation	HCHO, VOC, PM_2.5_, O_3_, NO_2_, temperature, RH
Sweden (2019), [48]	4 (preschool)	All seasons	Low emitting materials	Heat recovery ventilation & heat recovery with DCV	Temperature, RH, particle-size distribution, CO_2_, NO_2_, HCHO and TVOC
Sweden (2019), [90]	7 school (145 classrooms)	Summer & winter	NA	Mechanical ventilation with DCV and centralized air handling units	Temperature, CO_2_
Southern Italy (2019), [91]	12 (lower secondary schools)	Summer & winter	NA	Natural ventilation	Temperature, RH, CO_2_, NO_2_, PM_2.5_, biological pollutants in indoor dust (endotoxins and Der p 1)

## Data Availability

No new data were created or analyzed in this study. Data sharing is not applicable to this article.
